# A Three-Year Survey on the Worldwide Occurrence of Mycotoxins in Feedstuffs and Feed

**DOI:** 10.3390/toxins4090663

**Published:** 2012-09-12

**Authors:** Inês Rodrigues, Karin Naehrer

**Affiliations:** 1 BIOMIN Singapore Pte Ltd., 3791 Jalan Bukit Merah #08-08, E-Centre@Redhill, Singapore; 2 BIOMIN Holding GmbH, Industriestrasse 21, Herzogenburg 3130, Austria; Email: karin.naehrer@biomin.net

**Keywords:** aflatoxins, deoxynivalenol, fumonisins, zearalenone, ochratoxin A, mycotoxins, occurrence, survey

## Abstract

Between January 2009 and December 2011, a total of 7049 corn, soybean/soybean meal, wheat, dried distillers grains with solubles (DDGS) and finished feed samples were analyzed for the occurrence of aflatoxins (Afla), zearalenone (ZEN), deoxynivalenol (DON), fumonisins (FUM) and ochratoxin A (OTA). Samples were sourced in the Americas, Europe and Asia. Afla, ZEN, DON, FUM and OTA were present respectively in 33%, 45%, 59% 64% and 28% of analyzed samples between 2009 and 2011. From the 23,781 mycotoxin analyzes performed, 81% were positive for at least one mycotoxin. Results of this survey are provided by calendar year, in order to potentially show different trends on mycotoxin occurrence in distinct years: by commodity type and within the same commodity, and by region, to potentially reveal differences in mycotoxin contamination in commodities sourced in diverse regions.

## 1. Introduction

Mycotoxins refer to a diverse group of compounds produced by a wide range of different fungi, normally after a phase of balanced growth. Plant genetics, exposure to fungal spores, weather conditions and climate during planting, growing and harvesting, insect damage, crop management and use of fungicides, are some of the factors that influence the growth of fungi on crops and their subsequent mycotoxin production. Mycotoxin-producing fungi are commonly sub-divided into field fungi and storage fungi; however, the actual colonization and proliferation of fungi is not clear cut, but depends on the environmental and ecological circumstances, and the resulting toxins will differ accordingly. Moisture and temperature have a major influence on mold growth and mycotoxin production. Pathogenic fungi that invade crops prior to harvest usually require higher moisture levels (200–250 g/kg) for infection than fungi that can proliferate during storage (130–180 g/kg) [1].

Interestingly, the presence of mycotoxin-producing fungi in a plant is not always conducive to contamination with mycotoxins. In order for fungi to produce these secondary metabolites, they have to be stressed by some factor [2], such as nutritional imbalance, drought or water excess. 

This paper gathers information on the presence of mycotoxins in the commodities most commonly used for feed production and in finished feed through a period of 3 years. From January 2009 until December 2011, 23,781 mycotoxin analyses were performed on 7049 samples sourced in North and South America (the Americas), Europe and Asia. Middle Eastern and African samples were gathered in previously published reports [3,4] and were therefore excluded from this paper. Samples were analyzed for some or all mycotoxins: aflatoxins (Afla), zearalenone (ZEN), deoxynivalenol (DON), fumonisins (FUM) and ochratoxin A (OTA). Data is grouped for discussion as follows: per calendar year, in order to potentially show different trends in mycotoxin occurrence in distinct years; by commodity type and within the same commodity, and by region, to potentially reveal differences in mycotoxin contamination in commodities sourced in diverse regions. 

## 2. Results and Discussion

### 2.1. Results by Calendar Year

Table 1 reflects the contamination of all samples, regardless of their nature, for the sum of the three years, and then separated by calendar year. Afla, ZEN, DON, FUM and OTA were respectively present in 33%, 45%, 59%, 64% and 28% of analyzed samples between 2009 and 2011. Positive samples respectively averaged contamination levels of 63, 233, 1104, 1965 and 11 ppb for these mycotoxins. When data is separated by year, these values actually do not greatly differ. Within the three years of the study, the highest level of Afla was found in a corn sample from Vietnam (maximum: 6105 ppb); two wheat samples from Australia had the highest DON (maximum: 23,278 ppb) and ZEN (maximum: 49,307 ppb) levels found, while finished feed from China and Pakistan presented the highest levels of FUM (maximum: 77,502 ppb) and OTA (maximum: 1582 ppb), respectively. 

### 2.2. Results by Commodity and Region Sourced

Table 2, Table 3, Table 4, Table 5, Table 6, Table 7, Table 8, Table 9, Table 10, Table 11, Table 12 present data referring to mycotoxin contamination in different feedstuffs and finished feed, globally and separated by geographical region sourced. 

**Table 1:** Annual global trend regarding mycotoxin occurrence in corn, soybean meal (SBM), wheat, dried distillers grains with solubles (DDGS) and finished feed samples surveyed in the Americas, Europe and Asia.

	2009–2011					2009					2010					2011				
ALL SAMPLES	Afla	ZEN	DON	FUM	OTA	Afla	ZEN	DON	FUM	OTA	Afla	ZEN	DON	FUM	OTA	Afla	ZEN	DON	FUM	OTA
Number of tested samples	4,627	5,402	5,819	4,670	3,263	1,050	1,402	1,438	1,007	702	1,464	1,820	1,955	1,645	1,102	2,113	2,180	2,426	2,018	1459
Positive (%)	33	45	59	64	28	40	40	51	72	31	33	49	63	65	27	30	44	61	58	27
Average of positive (ppb)	63	233	1,104	1,965	11	82	198	794	2,467	16	45	244	1,304	1,906	7	64	243	1,093	1,711	11
Average (ppb)	21	104	651	1,249	3	33	79	408	1,767	5	15	120	816	1,245	2	19	108	662	994	3
median of positive (ppb)	9.0	80.0	505.0	958.0	2.0	11.0	87.0	496.0	1,094.0	3.0	8.0	85.0	540.0	983.0	2.3	9.0	71.0	486.0	849.0	2.0
1^st^ quartile of positive (ppb)	2.1	40.0	256.0	425.8	0.9	3.0	45.0	270.3	486.0	1.5	2.0	40.7	274.0	447.0	0.9	2.0	37.0	222.3	360.3	0.9
3^rd^ quartile of positive (ppb)	41.0	198.3	1,055.0	2,254.3	6.0	43.5	182.0	934	3,064	5.7	34.4	226.0	1,255.8	2,225.0	5.6	52.0	186.0	1,000.8	2,011.3	6.8
Maximum (ppb)	6,105	23,278	49,307	77,502	1,582	6,105	7,422	10,945	23,100	1,582	4,687	4,027	49,000	53,700	331	2,230	23,278	49,307	77,502	400
Commodity found	Corn	Wheat	Wheat	Finished feed	Finished feed	Corn	Corn	DDGS	Corn	Finished feed	Corn	Corn	Wheat	Corn	Wheat	Corn	Wheat	Wheat	Finished feed	Corn
Country of origin	Vietnam	Australia	Australia	China	Pakistan	Vietnam	Japan	Vietnam	Brazil	Pakistan	China	China	Austria	Brazil	Austria	Pakistan	Australia	Australia	China	India

**Table 2:** Mycotoxin occurrence in corn samples surveyed in North and South America and in Central and Southern Europe (no corn samples sourced in Northern Europe were surveyed).

	North America					South America					Central Europe					Southern Europe				
CORN	Afla	ZEN	DON	FUM	OTA	Afla	ZEN	DON	FUM	OTA	Afla	ZEN	DON	FUM	OTA	Afla	ZEN	DON	FUM	OTA
Number of tested samples	375	395	390	466	126	809	321	322	807	147	16	379	535	30	21	42	52	59	48	31
Positive (%)	26	29	79	39	10	25	43	17	92	12	31	39	72	60	10	36	21	47	90	29
Average of positive (ppb)	67	251	1,085	1,357	5	7	176	214	3,226	133	2	123	1,421	2,180	2	9	290	985	2,271	15
Maximum (ppb)	920	4,787	24,900	22,900	18	273	1,800	939	53,700	355	3	849	26,121	7,680	3	44	1,546	3,851	11,050	46
Average (ppb)	17	74	857	533	1	2	75	37	2,966	16	1	47	1,028	1,308	0	3	61	468	2,035	4
Median of positive (ppb)	10.1	86.4	565.0	490.0	2.3	1.8	87.1	172.0	2,008.0	71.3	1.5	78.5	716.0	684.0	2.4	4.0	166.0	523.0	1,407.0	9.3
1st quartile of positive (ppb)	2.6	59.9	300.0	280.0	1.4	1.0	40.4	140.0	859.5	20.0	1.4	42.3	431.5	276.3	2.2	1.6	73.5	308.5	756.0	1.5
3rd quartile of positive (ppb)	62.3	167.8	931.0	1,160.5	3.1	4.9	222.0	241.4	3,890.0	274.8	1.8	155.0	1,575.5	4,503.8	2.5	11.6	275.5	705.0	3,265.5	28.8

**Table 3:** Mycotoxin occurrence in corn samples surveyed in North, South-East and South Asia and in Oceania.

	North Asia					South-East Asia					South Asia					Oceania				
CORN	Afla	ZEN	DON	FUM	OTA	Afla	ZEN	DON	FUM	OTA	Afla	ZEN	DON	FUM	OTA	Afla	ZEN	DON	FUM	OTA
Number of tested samples	447	470	477	443	420	330	319	218	326	218	108	108	106	108	107	11	11	11	11	11
Positive (%)	12	67	92	75	10	71	20	45	83	12	82	9	22	74	27	18	27	27	64	9
Average of positive (ppb)	114	437	1,154	2,816	4	146	288	307	1,568	9	240	269	278	845	31	3	636	182	2,823	1
Maximum (ppb)	4,687	7,446	15,073	23,499	19	6,105	2,601	4,805	19,289	80	2,230	1,099	1,150	6,196	400	5	1,251	249	5,438	1
Average (ppb)	13	292	1,062	2,111	0	104	59	140	1,293	1	197	25	60	626	8	1	173	50	1,796	0
Median of positive (ppb)	7.0	176.0	640.0	1,518.5	1.4	38.0	97.0	182.0	1,033.0	3.0	96.0	78.5	190.0	541.0	7.4	3.0	626.0	179.0	2,344.0	1.2
1st quartile of positive (ppb)	2.0	63.9	309.5	592.5	0.7	11.0	51.0	103.5	552.0	0.75	13.0	67.3	104.0	293.3	2.0	2.0	328.0	148.5	1,453.0	1.2
3rd quartile of positive (ppb)	35.5	435.0	1,444.5	3,593.5	4.1	137.5	206.0	351.5	1,720.0	6.3	312.0	174.3	348.5	796.0	15.0	4.0	938.5	214.0	4,023.0	1.2

**Table 4:** Mycotoxin occurrence in soybean meal samples surveyed in North and South America and in Central and Southern Europe (no soybean meal samples sourced in Northern Europe were surveyed).

	North America					South America					Central Europe					Southern Europe				
SOYBEAN MEAL	Afla	ZEN	DON	FUM	OTA	Afla	ZEN	DON	FUM	OTA	Afla	ZEN	DON	FUM	OTA	Afla	ZEN	DON	FUM	OTA
Number of tested samples	74	50	45	46	18	60	53	55	60	51	8	31	43	2	3	23	23	25	21	22
Positive (%)	1	10	18	0	17	8	34	29	5	10	38	6	21	0	33	22	0	24	29	18
Average of positive (ppb)	2	83	1,007	-	4	1	129	208	230	5	1	36	470	-	21	2	-	419	1,017	1
Maximum (ppb)	2	144	5,500	-	6	1	807	428	315	10	1	56	741	-	21	3	-	908	5,088	1
Average (ppb)	0	8	179	-	1	0	44	61	12	0	0	2	98	-	7	0	-	101	291	0
Median of positive (ppb)	2.0	50.8	187.0	-	4.6	1.0	81.0	197.0	274.0	1.0	1.2	35.7	450.0	-	21.4	1.8	-	338.5	95.0	1.2
1st quartile of positive (ppb)	2.0	45.3	143.0	-	3.2	1.0	52.3	83.8	188.0	0.9	1.2	25.6	363.0	-	21.4	1.4	-	286.5	90.3	0.9
3rd quartile of positive (ppb)	2.0	142.4	733.8	-	5.2	1.0	130.3	291.8	294.5	10.4	1.3	45.9	494.0	-	21.4	2.7	-	428.0	510.8	1.3

**Table 5:** Mycotoxin occurrence in soybean meal samples surveyed in North, South-East and South Asia and in Oceania.

	North Asia					South-East Asia					South Asia					Oceania				
SOYBEAN MEAL	Afla	ZEN	DON	FUM	OTA	Afla	ZEN	DON	FUM	OTA	Afla	ZEN	DON	FUM	OTA	Afla	ZEN	DON	FUM	OTA
Number of tested samples	36	34	37	35	33	109	105	105	109	105	16	16	16	16	16	3	3	3	3	3
Positive (%)	6	35	38	6	24	22	15	18	4	16	63	0	31	0	56	67	33	33	0	33
Average of positive (ppb)	3	63	149	316	4	5	43	201	265	5	3	-	186	-	17	1	48	150	-	3
Maximum (ppb)	3	398	314	321	19	74	70	973	427	21	7	-	259	-	46	1	48	150	-	3
Average (ppb)	0	22	56	18	1	1	7	36	10	1	2	-	58	-	10	1	16	50	-	1
Median of positive (ppb)	2.8	31.3	107.0	316.0	1.6	1.0	38.0	155.0	227.5	2.4	2.0	-	249.0	-	14.4	1.0	48.0	150.0	-	3.1
1st quartile of positive (ppb)	2.7	25.3	96.55	313.5	0.9	1.0	34.8	87.5	207.5	0.9	1.0	-	90.0	-	1.7	1.0	48.0	150.0	-	3.1
3rd quartile of positive (ppb)	2.9	41.8	201.5	318.5	4.4	3.25	53.3	220.5	285.3	5.6	3.5	-	251.0	-	23.2	1.0	48.0	150.0	-	3.1

**Table 6:** Mycotoxin occurrence in wheat/wheat bran samples surveyed in North and South America.

	North America					South America				
WHEAT/BRAN	Afla	ZEN	DON	FUM	OTA	Afla	ZEN	DON	FUM	OTA
Number of tested samples	15	16	25	7	2	40	32	17	40	11
Positive (%)	20	13	76	0	50	3	47	53	5	45
Average of positive (ppb)	5	275	1,029	-	1	3	145	947	1,407	29
Maximum (ppb)	9	513	7,000	-	1	3	393	2,520	1,715	43
Average (ppb)	1	34	782	-	0	0	68	501	70	13
Median of positive (ppb)	4.1	274.5	600.0	-	0.8	2.6	72.5	906.3	1,407.0	32.9
1st quartile of positive (ppb)	2.8	155.3	300.0	-	0.8	2.6	41.2	505.0	1,253.0	29.2
3rd quartile of positive (ppb)	6.6	393.8	975.5	-	0.8	2.6	232.0	1,005.6	1,561.0	39.0

**Table 7:** Mycotoxin occurrence in wheat/wheat bran samples surveyed in Northern, Central and Southern Europe.

	Northern Europe					Central Europe					Southern Europe				
WHEAT/BRAN	Afla	ZEN	DON	FUM	OTA	Afla	ZEN	DON	FUM	OTA	Afla	ZEN	DON	FUM	OTA
Number of tested samples	1	71	71	1	2	13	256	436	9	22	14	17	24	10	13
Positive (%)	0	15	55	0	0	31	12	55	33	23	43	0	38	30	8
Average of positive (ppb)	-	109	1,058	-	-	2	89	1,534	268	69	2	-	1,204	386	1
Maximum (ppb)	-	233	7,341	-	-	2	336	49,000	450	331	6	-	3,505	925	1
Average (ppb)	-	17	581	-	-	0	10	848	89	16	1	-	452	116	0
Median of positive (ppb)	-	96.0	641.0	-	-	1.6	65.0	514.0	246.0	3.8	1.6	-	716.0	151.0	0.7
1st quartile of positive (ppb)	-	62.5	442.0	-	-	1.2	47.3	361.0	177.5	2.8	1.4	-	503.0	117.0	0.7
3rd quartile of positive (ppb)	-	131.0	932.5	-	-	2.0	122.8	960.0	348.0	5.4	1.8	-	1,864.0	538.0	0.7

**Table 8:** Mycotoxin occurrence in wheat/wheat bran samples surveyed in North and South-East Asia and in Oceania (no wheat/bran samples sourced in South Asia were surveyed).

	North Asia					South-East Asia					Oceania				
WHEAT/BRAN	Afla	ZEN	DON	FUM	OTA	Afla	ZEN	DON	FUM	OTA	Afla	ZEN	DON	FUM	OTA
Number of tested samples	76	72	75	73	67	40	40	40	40	40	109	115	109	109	108
Positive (%)	7	42	87	11	22	3	40	65	5	30	5	28	48	12	8
Average of positive (ppb)	6	74	922	371	2	1	531	2,251	172	6	3	1,546	5,046	269	2
Maximum (ppb)	20	465	5,331	874	7	1	6,641	41,439	292	30	7	23,278	49,307	1,196	4
Average (ppb)	0	31	799	41	0	0	212	1,463	9	2	0	430	2,407	32	0
Median of positive (ppb)	3.3	47.7	426.0	297.5	1.0	1	52.5	198.5	172.0	3.9	2.0	179.5	719.0	196.0	1.6
1st quartile of positive (ppb)	1.0	20.6	168.0	191.5	0.7	1	44.8	97.0	112.0	1.4	2.0	76.8	90.0	120.0	1.0
3rd quartile of positive (ppb)	3.6	82.1	1,279.0	471.3	2.0	1	216.8	1,483.3	232.0	5.7	5.0	351.0	5,870.3	216.0	3.7

**Table 9:** Mycotoxin occurrence in DDGS samples surveyed in North America, North and South-East Asia and in Oceania.

	North America					North Asia					South-East Asia					Oceania				
DDGS	Afla	ZEN	DON	FUM	OTA	Afla	ZEN	DON	FUM	OTA	Afla	ZEN	DON	FUM	OTA	Afla	ZEN	DON	FUM	OTA
Number of tested samples	42	80	80	62	24	68	71	76	58	61	22	22	22	22	22	10	10	10	10	10
Positive (%)	29	80	96	84	33	21	85	93	62	39	0	100	100	100	41	0	10	20	20	20
Average of positive (ppb)	7	194	2,186	1,329	2	54	321	3,068	1,596	6	-	286	3,618	1,481	2	-	51	1,318	2,138	5
Maximum (ppb)	14	849	10,100	6,400	4	340	2,319	15,597	9,782	26	-	1,179	19,096	8,449	4.2	-	51	2,577	2,837	6
Average (ppb)	2	156	2,104	1,115	1	11	272	2,866	991	3	-	286	3,618	1,481	1	-	5	264	428	1
Median of positive (ppb)	6.6	138.5	1,520.0	840.0	1.4	34.0	171.0	2,390.0	490.0	3.1	-	163.5	2,127.5	848	1.8	-	51	1,317.5	2,138.0	5.0
1st quartile of positive (ppb)	4.3	95.7	960.0	462.0	1.2	21.1	56.6	1,152.5	242.3	1.6	-	86.0	970.3	419.3	0.8	-	51	687.8	1,788.5	4.5
3rd quartile of positive (ppb)	9.4	222.9	2,600.0	1,534.5	2.3	43.1	389.0	4,212.0	1,421.3	8.5	-	313.5	4,570.0	1,923.0	3.6	-	51	1,947.3	2,487.5	5.5

**Table 10:** Mycotoxin occurrence in finished feed samples surveyed in North and South America.

	North America					South America				
FINISHED FEED	Afla	ZEN	DON	FUM	OTA	Afla	ZEN	DON	FUM	OTA
Number of tested samples	21	42	55	32	8	203	119	130	224	49
Positive (%)	24	52	65	47	0	26	57	13	94	12
Average of positive (ppb)	29	299	1,718	2,369	-	6	201	250	1,665	26
Maximum (ppb)	56	1,710	6,100	11,400	-	83	3,570	808	10,380	49
Average (ppb)	7	157	1,125	1,111	-	2	115	33	1,569	3
Median of positive (ppb)	23.8	161.5	1,350.0	1,000.0	-	2.9	89.6	204.0	1,142.0	25.1
1st quartile of positive (ppb)	10.4	122.0	600.0	500.0	-	1.3	23.1	179.4	610.0	16.4
3rd quartile of positive (ppb)	52.0	198.0	2,200.0	3,150.0	-	5.5	164.8	283.0	2,087.5	32.2

**Table 11:** Mycotoxin occurrence in finished feed samples surveyed in Northern, Central and Southern Europe.

	Northern Europe					Central Europe					Southern Europe				
FINISHED FEED	Afla	ZEN	DON	FUM	OTA	Afla	ZEN	DON	FUM	OTA	Afla	ZEN	DON	FUM	OTA
Number of tested samples	1	27	27	1	1	45	489	579	65	95	66	72	104	48	51
Positive (%)	0	37	74	0	0	2	48	67	40	37	47	18	37	75	53
Average of positive (ppb)	-	87	641	-	-	1	118	792	327	4	6	72	431	2,007	2
Maximum (ppb)	-	339	1,889	-	-	1	1,045	25,759	2,282	30	103	165	1,252	7,008	17
Average (ppb)	-	32	475	-	-	0	56	533	131	2	3	13	158	1,505	1
Median of positive (ppb)	-	44.5	449.5	-	-	0.8	70.0	509.3	135.5	2.7	2.4	61.0	336.0	1,797.0	0.8
1st quartile of positive (ppb)	-	41.0	203.3	-	-	0.8	34.0	269.3	60.1	1.3	1.2	40.0	224.5	554.0	0.7
3rd quartile of positive (ppb)	-	65.3	914.5	-	-	0.8	117.0	917.0	341.5	6.0	4.2	88.0	515.3	3,040.3	1.0

**Table 12:** Mycotoxin occurrence in finished feed samples surveyed in North, South-East, South Asia and in Oceania.

	North Asia					South-East Asia					South Asia					Oceania				
FINISHED FEED	Afla	ZEN	DON	FUM	OTA	Afla	ZEN	DON	FUM	OTA	Afla	ZEN	DON	FUM	OTA	Afla	ZEN	DON	FUM	OTA
Number of tested samples	622	661	671	604	575	465	454	447	465	448	127	120	111	123	122	75	86	86	74	74
Positive (%)	20	79	89	67	32	81	66	35	71	42	95	49	22	71	93	9	26	34	14	19
Average of positive (ppb)	24	271	829	1,542	4	29	53	287	800	3	95	51	156	438	23	5	291	296	1326	9
Maximum (ppb)	225	5,791	19,141	77,502	60	431	253	2,683	22,693	36	2,454	168	634	1,507	1,582	9	926	709	3,229	41
Average (ppb)	5	213	741	1,026	1	23	35	102	566	1	91	25	34	310	21	0	74	100	179	2
Median of positive (ppb)	11.0	106.0	513.5	806.0	2.0	13.0	41.0	143.5	570.0	1.5	49.0	40.0	128.0	336.0	4.0	3.0	128.0	224.0	1,283.0	5.2
1st quartile of positive (ppb)	3.1	47.0	246.5	366.0	0.9	4.0	31.0	91.0	332.0	0.7	20.0	31.0	72.8	216.0	2.1	1.5	37.3	148.0	409.0	1.9
3rd quartile of positive (ppb)	30.6	294.5	920.0	1,758.0	4.4	36.0	58.0	266.0	970.0	3.1	100	58.5	173.25	629	7.2	8.5	447.8	432.0	1,939.0	9.3

The corn contamination pattern differed between regions (Table 2 and Table 3). Both in North America and in Central Europe the main contaminant of corn was shown to be DON (79% and 72% of positive samples, respectively), followed by FUM in both cases. Average contamination levels were quite similar for both regions (North America average of positive: 1085 ppb; Central Europe average of positive: 1421 ppb). In South America and southern Europe, the main contaminant observed in corn was FUM (92% and 90% of positive samples, respectively). Average contamination levels in both regions were different, with South American samples presenting higher values (average of positive: 3226 ppb) in comparison with Southern Europe (average of positive: 2271 ppb). The pattern followed in corn sourced in North Asia followed the same than that of North America and Central Europe with DON being the main contaminant (present in 92% of tested samples) at average levels of 1154 ppb. In Asia, in equatorial regions, the presence of Afla increased dramatically with 82% of positive samples for corn sourced in South Asia and 71% of positive samples for corn sourced in South-East Asia. Nonetheless, as the data show, the presence of fusariotoxins, such as FUM and DON, in these regions cannot be ignored. Corn samples sourced in Oceania presented quite high average levels of FUM (average of positive: 2823 ppb) present in 64% of analyzed samples. 

In comparison with corn, soybean meal (SBM) appears to be less susceptible to mycotoxin contamination (Table 4 and Table 5). In general, it can be said that mycotoxins such as ZEN and DON occur most frequently in this commodity throughout all regions. Surprisingly, one sample sourced in the USA presented a contamination level of 5500 ppb DON and another sample sourced in Turkey was positive for FUM with a contamination of 5088 ppb. This shows that even this commodity might unexpectedly present high contamination levels when conditions are favorable for mycotoxin production.

According to data presented in Table 6, Table 7, Table 8, the major contaminant of wheat throughout all regions was DON. ZEN was also shown to be a main contaminant of this commodity, which is not a great surprise, as it is commonly known to co-occur with DON, a mycotoxin that shares the same producing fungi. In regards to positive samples, average levels for the American region (North and South America), Northern and Southern Europe and North Asia were around the 1000 ppb. In Central Europe, South-East Asia and Oceania, DON average levels were higher than that with 1534 ppb, 2251 ppb and 5046 ppb, respectively. A wheat sample from Austria was analyzed in October 2010 and showed a contamination of 49,000 ppb DON. Two distinct wheat samples from Australia were contaminated with 49,307 ppb DON and 23,278 ppb ZEN, the maximum levels found for this commodity worldwide. Both samples were analyzed in April 2011. Actually, the mycotoxin levels registered in both Austria in the year 2010 and Australia in the year 2011 were much higher than those reported for previous years [4]. As there is reason to believe that climate change can affect infection of crops with toxigenic fungi, the growth of these fungi and the production of mycotoxins [5], it is perhaps not erroneous to speculate that the occurrence of heavy rain and floods in both countries prior to crop harvest were responsible for such contamination levels. 

In regards to Dried Distillers Grain with Solubles (DDGS), DON, ZEN and FUM were generally the main contaminants. Data shown in Table 9 reveals very high average levels for contaminated samples, especially for DON. These results reiterate those published in a previous report [6], thus confirming the need for monitoring the mycotoxin content of DDGS prior to its inclusion in animal diets. 

Data shown in Table 10, Table 11, Table 12 pertain to finished feeds (swine, poultry and dairy). The contamination pattern observed for each region can be related to that of the typically used main feedstuff. For example, in North American diets, corn is the main ingredient used, which explains to a great extent the prevalence of DON in these feeds. The same situation is observed in South America, where FUM is the main contaminant of corn, thus the major mycotoxin present in finished diets. In northern European countries, finished feeds typically have a higher proportion of cereals such as wheat, thus the matching pattern between wheat and finished feed sourced in this region. Besides this obvious but interesting conclusion, from an animal health and performance point of view, it is important to reiterate the fact that incredibly high maximum levels were found in finished feed samples sourced in all regions, but especially in Asia (2454 ppb Afla in South Asia, 5791 ppb ZEN, 19,141 ppb DON and 77,502 ppb FUM in North Asia and 1582 ppb OTA in South Asia). This draws attention to the fact that animals will frequently be faced with peak and fluctuating mycotoxin levels. Besides being well above regulated and recommended EU-values for the presence of mycotoxins in animal feed [7,8,9], the extremely high mycotoxin levels found will greatly impact performance and the health of animals ingesting them. 

### 2.3. Co-occurrence of Mycotoxins

The simultaneous exposure of animals and poultry to more than one toxin is of concern and requires more study [10]. Synergistic effects may explain why animals sometimes respond negatively to mycotoxin levels much lower than those reported in scientific studies as able to cause mycotoxicoses. 

From the 7049 samples, only 19% of them tested negative for the presence of the five analyzed mycotoxins. 33% showed the presence of one of them and two or more of the tested mycotoxins were present in 48% of the commodities. 

Interestingly, when co-occurrence is evaluated in finished feed from different regions, differences are obvious. 10% of finished feeds in the Americas tested below the limit of detection for all analyzed mycotoxins, 50% tested positive for the presence of one mycotoxin, and in 40% of the samples, two or more mycotoxins were present. In Europe, 39% of the finished feed samples analyzed tested positive for 2 or more mycotoxins, 37% tested positive for one mycotoxin and 24% tested negative (below the limit of detection) for all five mycotoxins. In Asia, multi-mycotoxin contamination seems to be more prevalent, as 82% of the finished feed samples tested positive for the presence of two or more mycotoxins and 12% showed to be contaminated with one mycotoxin. Only 6% of the tested samples were found to be below the limits of detection. 

## 3. Experimental Section

Between January 2009 and December 2011, a total of 7049 corn, soybean/soybean meal, wheat, dried distillers grains with solubles and finished feed samples were analyzed for the occurrence of aflatoxins, zearalenone, deoxynivalenol, fumonisins and ochratoxin A. The number of analyzed samples totaled 1653 in 2009, 2303 in 2010 and 3093 in 2011. Samples were sourced directly at animal farms or animal feed production sites from three major regions (Americas, Europe and Asia-Pacific). From the total number of samples analyzed throughout the 3 years, 2054 samples were sourced in North (12.5%) and South (17.3%) America; 2151 samples were sourced in Europe (Northern: 1.5%; Central: 23.9%; Southern: 3.5%) and 2844 samples were sourced in Asia (North: 20.3%; South-East: 14.0%; South: 3.7% and Oceania: 3.3%). Samples from North America comprise those from the United States of America (only 1 sample originated in Canada was analyzed). 93% of the samples included in the South America group were sourced in Brazil with the remaining 7% originating from Argentina and Paraguay. European samples were sourced in the following countries: Norway, Sweden, Finland, Ireland and United Kingdom (Northern Europe); Austria, Belgium, Germany, France, the Netherlands, Hungary, Romania, Slovakia, Slovenia, Poland, Ukraine, Czech Republic, Croatia, Lithuania, Ukraine, Russia and Belorussia (Central Europe) and Greece, Italy, Portugal, Spain, Turkey and Serbia (Southern Europe). Samples in the group North Asia were sourced in China (83%), Japan, Korea and Taiwan. Malaysia, Philippines, Thailand, Vietnam and Indonesia comprised the South-East Asia group. Samples sourced in Sri Lanka, Pakistan, Bangladesh and India were grouped as South Asia and those from New Zealand and Australia in the Oceania group.

Analytical personnel and/or laboratory staff were not involved, and therefore were not able to influence any part of the sampling procedure. However, good sampling methods [11] were explained and sample providers were advised to follow them. Samples received in the lab weighed approximately 1 kg. After grinding the full lot sample, a subsample was taken for the actual analytical process. For dry and low-fat containing samples, such as various raw cereals, a ROMER Series II^®^ subsampling mill (Romer Labs^®^ Diagnostic GmbH, Tulln, Austria) was used. For all other commodities and ready-ground samples, kitchen blenders or other adequate instruments were used for homogenization. A choice could be made regarding the mycotoxins to be analyzed for, either “full toxin screen”, which covered aflatoxins (a sum of aflatoxin B1, aflatoxin B2, aflatoxin G1 and aflatoxin G2 content), zearalenone (ZEN), deoxynivalenol (DON), fumonisins (a sum of fumonisin B1 and fumonisin B2), and ochratoxin A (OTA), or analyses of selected mycotoxins. This explains why the number of analyzed mycotoxins in certain regions is sometimes different depending on the specific mycotoxin. The origin (name and location of submitter) of samples was kept strictly confidential; analytical certificates were submitted only to the originators of samples. Eighty percent of the samples were analyzed by high performance liquid chromatography (HPLC). Twenty percent of the samples were analyzed by Enzyme-Linked Immunosorbent Assay (ELISA), but only in North America and Europe where this option was available. Only single commodities were analyzed by ELISA. All analyses were done in fully accredited service labs. More complex matrixes that could interfere with the ELISA method, such as DDGS and finished feed, were analyzed by HPLC. Besides the nature of the samples, the geographical availability of analytical methods also explains the reason why certain feedstuffs were analyzed by ELISA rather than HPLC. Mycotoxin analyses were carried out as published by Rodrigues and Naehrer [4]. For the purpose of data analysis, non-detect levels are based on the detection limits (LOD) of the test method for each toxin (Table 13).

**Table 13:** Limit of detection of applied analytical methods.

	HPLC	ELISA
Afla (sum of AfB_1_, AfB_2_, AfG_1_, AfG_2_)	-	1
AfB_1_, AfB_2_, AfG_1_, AfG_2_	0.3, 0.1, 0.1, 0.1	-
ZEN	10	40
DON	50	250
FB_1_ and FB_2_	25, 25	-
FUM (sum of FB_1_ and FB_2_)	-	250
OTA	0.2	2

## 4. Conclusions

The results of this three-year mycotoxin survey reiterate the importance of mycotoxin testing prior to the feeding of animals. From the 23,781 mycotoxin analyses performed on the major five mycotoxins in terms of animal health and performance, 81% were positive for at least one mycotoxin. The presence of more than one mycotoxin in approximately half of the samples draws attention to the multi-mycotoxin contamination of feedstuffs and feeds and to the synergistic effects of mycotoxins in animals. 

Moreover, it was shown that corn is a preferred substrate for fungal growth and mycotoxin production in comparison with soybean and wheat; however, seasons with abnormal weather conditions, such as excessive rain, have a great impact in contamination levels as shown with post-flooding wheat crops from Austria and Australia. DDGS continues to be a feedstuff which exhibits quite high contamination levels and for which careful screening prior to animal feeding is advised. 

For further studies, it would be interesting to understand the importance of the so-called masked mycotoxins to the overall contamination of feedstuffs and feed; nonetheless, this might be difficult, as these compounds cannot be detected with conventional analytical procedures.
